# Age- and calorie-independent life span extension from dietary restriction by bacterial deprivation in *Caenorhabditis elegans*

**DOI:** 10.1186/1471-213X-8-49

**Published:** 2008-05-05

**Authors:** Erica D Smith, Tammi L Kaeberlein, Brynn T Lydum, Jennifer Sager, K Linnea Welton, Brian K Kennedy, Matt Kaeberlein

**Affiliations:** 1 Department of Pathology, University of Washington, Seattle, WA 98195, USA; 2 Department of Biochemistry, University of Washington, Seattle, WA 98195 USA

## Abstract

**Background:**

Dietary restriction (DR) increases life span and delays age-associated disease in many organisms. The mechanism by which DR enhances longevity is not well understood.

**Results:**

Using bacterial food deprivation as a means of DR in *C. elegans*, we show that transient DR confers long-term benefits including stress resistance and increased longevity. Consistent with studies in the fruit fly and in mice, we demonstrate that DR also enhances survival when initiated late in life. DR by bacterial food deprivation significantly increases life span in worms when initiated as late as 24 days of adulthood, an age at which greater than 50% of the cohort have died. These survival benefits are, at least partially, independent of food consumption, as control fed animals are no longer consuming bacterial food at this advanced age. Animals separated from the bacterial lawn by a barrier of solid agar have a life span intermediate between control fed and food restricted animals. Thus, we find that life span extension from bacterial deprivation can be partially suppressed by a diffusible component of the bacterial food source, suggesting a calorie-independent mechanism for life span extension by dietary restriction.

**Conclusion:**

Based on these findings, we propose that dietary restriction by bacterial deprivation increases longevity in *C. elegans *by a combination of reduced food consumption and decreased food sensing.

## Background

Dietary restriction (DR), also referred to as calorie restriction, is an intervention that extends life span and delays the onset of age-related phenotypes in nearly all eukaryotic organisms in which it has been tested [1]. Simplistically, it is defined as a significant reduction in dietary intake in the absence of malnutrition. Many different approaches can be used to achieve DR. In mice and rats for example, life span extension is observed by either reducing the amount of food consumed daily (compared to an *ad libitum *control group) or by imposing an intermittent fasting regimen [2,3].

In addition to simply reducing the amount of food intake, the effects of altering dietary composition has also been examined in different organisms. Methionine-restricted mice [4] and rats [5] have an extended life span, suggesting that the nutritional composition of the diet can influence longevity in mammals. In fruit flies, reducing either the yeast extract or sugar composition of the food supply extends life span, although yeast extract appears to have the greatest impact on longevity [6]. Restriction of amino acids also increases life span in flies [7]. In yeast, reducing either the amount of glucose or amino acids in the growth media increases replicative life span [8-10].

Multiple methods for DR have been used in *C. elegans*, as in other model organisms [11]. One commonly used method in *C. elegans *is a genetic model, mutation of *eat-2*. The *eat *mutants were originally identified in a screen for defects in feeding behavior – they have reduced food consumption due to pharyngeal pumping defects [12,13]. Several alleles of *eat-2 *(and other *eat *mutants) increase life span, with longevity generally correlating with the degree to which pumping rate is decreased [14]. Life span extension from mutation of *eat-2 *is independent of the FOXO-family transcription factor DAF-16 and is additive with a longevity-enhancing allele of the insulin/IGF-1-like receptor *daf-2 *[14]. This has led to the generally accepted model that DR acts to modulate longevity in a genetic pathway distinct from insulin/IGF-1-like signaling (IIS) [15,16]. As with any genetic model of DR, however, *eat *mutants are not suitable for certain studies. The relative activity associated with different *eat-2 *alleles has not been completely characterized, so it is unclear whether life span extension from DR is maximized in studies using these alleles. In addition, *eat *mutants are food restricted from hatching, and DR during development may have secondary effects on adult physiology that are not fully understood.

DR by reducing food availability also increases life span in *C. elegans*. The standard method for culturing *C. elegans *in the laboratory is to maintain the nematodes on the surface of nematode growth medium (NGM) nutrient agar with *E. coli *OP50 as the food source. DR on NGM nutrient agar has been achieved by reducing the amount of peptone in the media so as to limit bacterial growth [17] or by reducing the amount of live bacteria present on the surface of the NGM nutrient agar [18]. Two methods of DR have also been described using non-standard, liquid-based growth conditions: bacterial dilution in S basal medium and axenic growth [19-23]. Both liquid-based methods increase life span relative to animals fed a diet of *E. coli *OP50 in S basal, and axenic growth behaves similarly to mutation of *eat-2 *in epistasis experiments with IIS [24]. Nonetheless, neither method is widely used for aging studies. This may be due to the potential differences in growth under standard conditions on an agar surface versus growth in liquid culture. Axenic growth is reported to cause delayed development and poor growth [22,25], and feeding animals *E. coli *in S basal shortens life span relative to growth on NGM agar [19]. In addition, DR during development has been shown to be sub-optimal, since animals switched to DR just before adulthood are longer-lived than those maintained on DR from hatching [26].

Recently, two groups independently reported an novel reduced bacterial feeding DR protocol carried out under standard conditions [27,28]. By measuring adult life span as a function of *E. coli *food concentration, both studies determined that complete removal of bacterial food early in adulthood (BD, bacterial food deprivation; also referred to as dietary restriction through food deprivation or dietary deprivation) optimally increases median and maximum life span. BD differs from previous studies describing DR by axenic growth in the culture method (solid versus liquid media) and the time of initiation (reproductively mature adults versus hatchlings)[27]. Although bacterial food is completely absent during adulthood in the BD regimen, BD-treated animals do not suffer from malnutrition (as evidenced by their increased life span and stress resistance [27,28]), thus meeting the commonly accepted definition for DR. Life span extension from BD has been recently validated in multiple wild-derived *C. elegans *strains, as well as in a second closely related nematode species *C. remanei *[29].

Like mutation of *eat-2 *or axenic growth [14,24], BD appears to modulate longevity by a mechanism distinct from reduced IIS [27,28]. While BD results in a more robust life span increase than mutation of *eat-2*, *eat-*2 mutants subjected to BD are not longer-lived than wild type animals on BD [27,28]. Taken together, these observations indicate that BD and mutation of *eat-2 *are likely to increase life span by similar or overlapping mechanisms. Thus, BD represents a simple plate-based method for DR that maximizes longevity from food restriction under standard nematode growth conditions and does not require additional genetic manipulation.

In order to better characterize the mechanism by which BD slows aging in *C. elegans*, we determined the effect of BD on survival as a function of the age at which BD is initiated. Previously, our group and an independent study [27,28] reported that BD increases life span in *C. elegans *when initiated at the last stage of larval development (L4) through day 11 of adulthood. Here, we expand this analysis to more advanced ages, after which mortality has become significant in the population. Similar to prior observations in *Drosophila *[30] and in mice [31], we find that BD enhances survival even when imposed late in life. Transient exposure to BD confers a life span benefit that correlates with stress resistance, as measured by thermotolerance. We further demonstrate that age-independent life span extension in response to BD is not solely due to reduced caloric intake, but is also influenced by a diffusible, cell-free component of the bacterial diet.

## Results and Discussion

**Figure 1:**
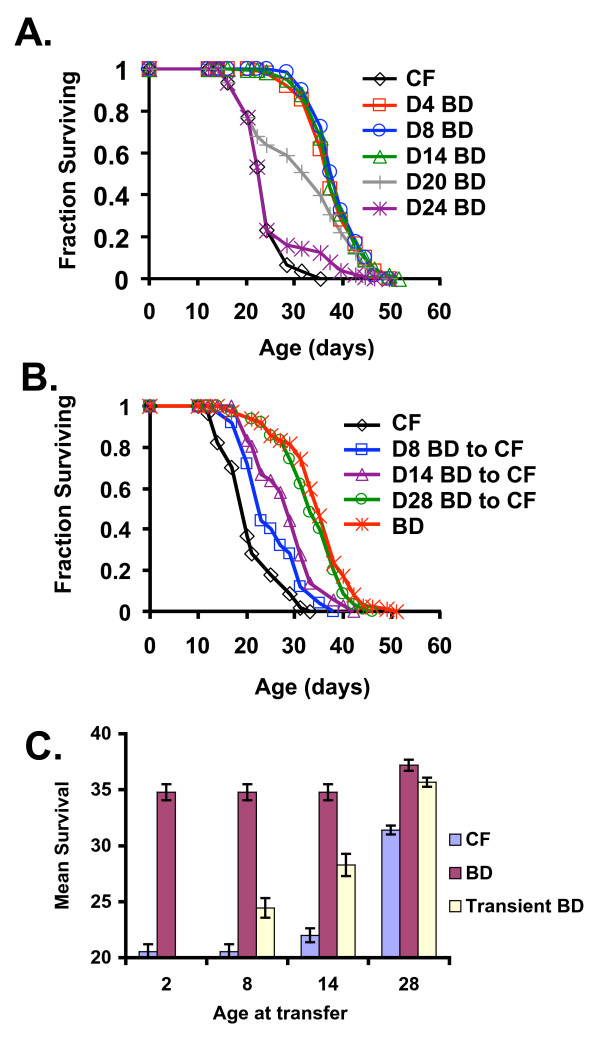
**Dietary restriction by bacterial food deprivation (BD) extends life span independent of age or duration**. **(A) **BD increases survival when initiated at 4 (red line, square symbols), 8 (blue line, circles), 14 (green line, triangles), 20 (gray line, plus symbols), or 24 (purple line, astericks) days of adulthood, relative to control fed (CF) animals. Mean life spans ± standard error, statistical significance, and number of animals examined provided in Table 1. **(B) **Animals subjected to transient BD, from the day 2 of adulthood to day 8 (blue line, square symbols), 14 (purple line, triangles), or 28 (green line, circles) have increased survival relative to animals maintained on CF until death (black line, diamonds). Mean life spans ± standard error, statistical significance, and number of animals examined provided in Table 2. (**C**) Mean survival of animals in (B) alive when BD-treated animals were transferred back to control fed conditions shows a significant increase in survival of animals exposed to transient BD relative to animals maintained on a control fed diet for life.

To determine the relationship between BD and age, animals were switched from control fed to BD diets, or vice versa, at different ages, and survival was monitored. A significant increase in survival was observed when animals were transferred to BD at day 4, 8, 14, 20, or 24 of adulthood (Figure 1A, Table 1). This was true even when BD was initiated after more than 50% of the control-fed population had died. Animals placed on BD at the 2^nd ^day of adulthood and returned to a control-fed diet at the 8^th^, 14^th^, or 28^th ^day of adulthood lived longer than animals fed a control diet for their entire life (Figure 1B, Table 2). By considering the survival of only those animals alive at the time of return to control fed conditions, it was apparent that transient BD induced a significant long-term protective effect resulting in enhanced survival after return to a control fed diet (Figure 1C). This is demonstrated by the significant increase in survival following return to a control fed diet, relative to animals maintained on a control fed diet for life.

**Table 1:** BD increases life span independent of the age at which it is initiated.

**Cohort**	**Number of animals**	**Mean Survival After Transfer**	**Mean Control Fed Survival After Transfer**	***p*-value**
CF	30	-	23.6 ± 0.7	-
Day 4 BD	110	37.6 ± 0.5	23.6 ± 0.7	1.4 × 10^-15^
Day 8 BD	79	38.6 ± 0.5	23.6 ± 0.7	2.1 × 10^-15^
Day 14 BD	158	38.1 ± 0.4	23.6 ± 0.7	4.8 × 10^-17^
Day 20 BD	112	35.3 ± 0.7	25.0 ± 0.7	9.1 × 10^-8^
Day 24 BD	52	35.2 ± 0.8	29.7 ± 1.2	0.01

'Mean survival after transfer' is the average life span of animals in each group that were alive at the time the cohort was switched from control fed (CF) to bacterial deprivation (BD). 'Mean control fed survival after transfer' is the average life span of control fed animals that were alive at the time the corresponding cohort was switched to BD. Survival data are shown as mean ± standard error. *P*-values were calculated by the Wilcoxon Rank-Sum test using the MATLAB 'ranksum' function.

**Table 2:** Transient BD has long-term survival benefits.

**Cohort**	**Number of animals**	**Mean Survival After Transfer**	**Mean Control Fed Survival After Transfer**	***p*-value**
CF	57	-	20.5 ± 0.6	-
BD	97	34.8 ± 0.7	20.5 ± 0.6	1.7 × 10^-19^
Day 8 BD to CF	50	24.4 ± 0.9	20.5 ± 0.6	3.0 × 10^-4^
Day 14 BD to CF	36	28.3 ± 1.0	22.0 ± 0.6	2.0 × 10^-6^
Day 28 BD to CF	117	35.7 ± 0.4	31.4 ± 0.4	0.03

'Mean survival after transfer' is the average life span of animals in each group that were alive at the time the cohort was switched from BD back to a control fed (CF) diet. 'Mean control fed survival after transfer' is the average life span of control fed animals that were alive at the time the corresponding cohort was switched to BD. Survival data are shown as mean ± standard error. *P*-values were calculated by the Wilcoxon Rank-Sum test using the MATLAB 'ranksum' function.  Bacterial deprivation (BD) was initiated at the 2^nd ^day of adulthood.

**Figure 2:**
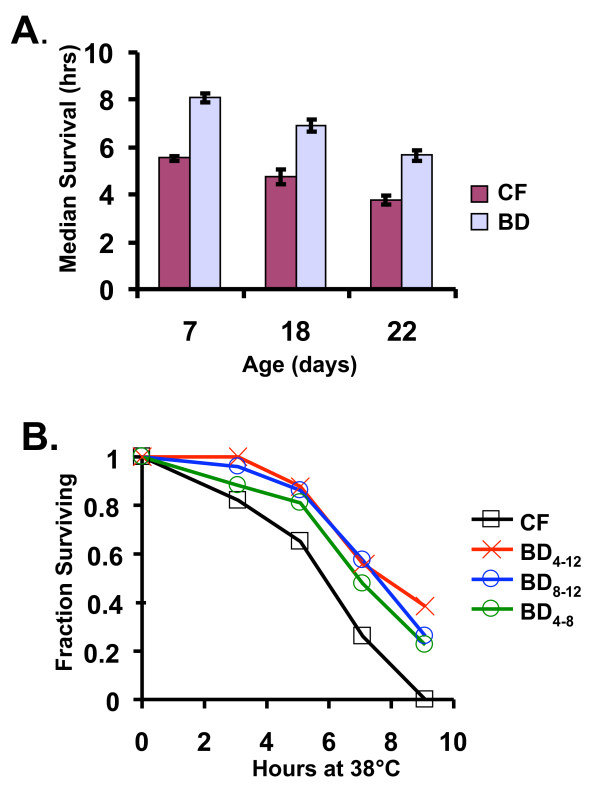
**Increased thermotolerance from BD is independent of age and maintained upon return to a control diet**. **(A) **BD increases survival at 38°C, relative to control fed (CF) animals at 7, 18, or 22 days of age. Error bars are standard error of the mean. **(B) **Thermotolerance (survival at 38°C) is significantly increased at the 12th day of adulthood relative to CF animals, regardless of whether BD is initiated at the 4th (BD_4–12_) or 8th (BD_8–12_) day of adulthood. Transient BD from the 4th to the 8th day of adulthood (BD_4–8_) also results in enhanced thermotolerance relative to CF animals.

We have previously reported that BD induces a robust increase in thermotolerance [27]. We wanted to determine whether worms exposed to transient BD retain this stress resistant phenotype, in correlation with the life span benefit. We observed an age-dependent decrease in thermotolerance of both control fed and BD animals; however, BD significantly increased thermotolerance at every age-point examined (Figure 2A; day 7, *p *= 4.3 × 10^-16^; day 18, *p *= 3.9 × 10^-7^; day 22, *p *= 1.7 × 10^-7^), consistent with prior observations [28]. Interestingly, enhanced thermotolerance was also maintained in animals subjected to transient BD (four days on BD then returned to the control diet for four days) (Figure 2B; *p *= 0.02), demonstrating that transient BD provides a long-term enhancement of thermotolerance as well as survival.

During our analysis of BD and the age at which it is initiated, we noted that older control fed animals qualitatively consumed less food than young animals, based on the rate at which bacterial food disappeared from the surface of the agar plates. This is consistent with prior reports that the rate of pharyngeal pumping, which is necessary for food to enter the intestine, decreases dramatically with age [20,32,33]. In a longitudinal study of age-related phenotypes, it was shown that no significant pumping can be measured in the last quartile of the mean life span [32]. This suggested to us the possibility that BD might increase life span when initiated in animals that were no longer consuming a substantial amount of bacterial food.

**Figure 3:**
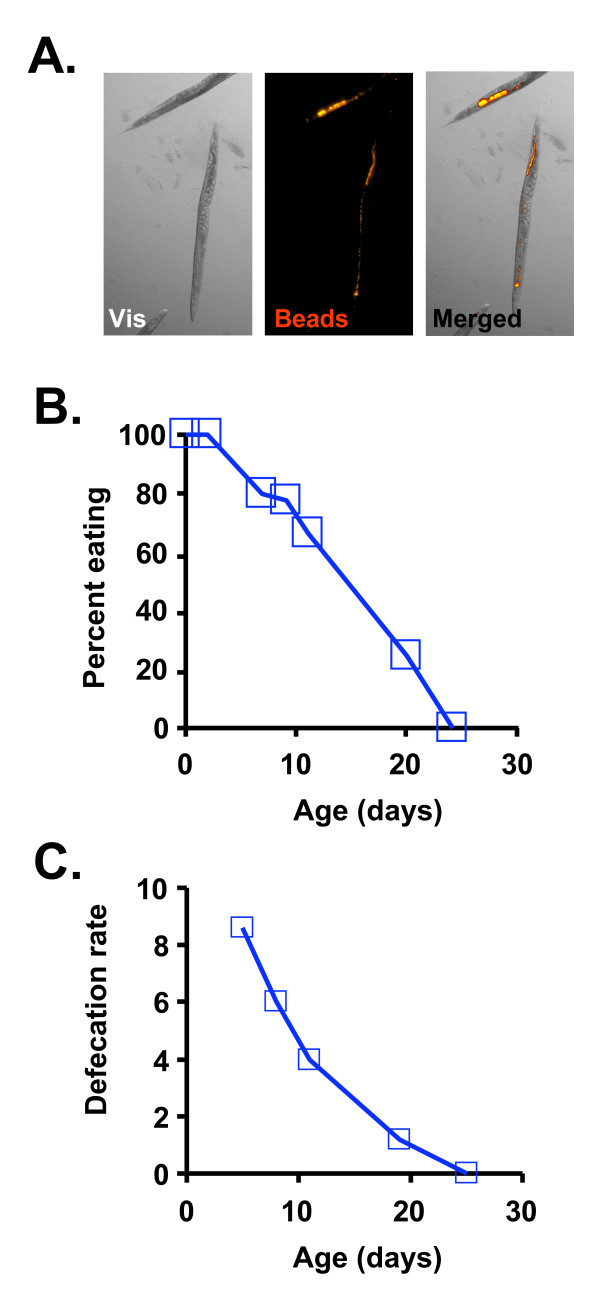
**Bacterial food consumption decreases with age**. **(A) **Fluorescent beads similar in size to bacterial cells are eaten and can be visualized in the intestines of young (day 4 adult) animals (Vis = visible light). **(B) **The percent of animals consuming a detectable quantity of fluorescent beads decreases with age in control fed (CF) animals. (**C) **The rate of defecation (average number of defecation cycles per animal in 10 minutes of observation) decreases with age.

To assay feeding behavior as a function of age, we quantified both food consumption and defecation. Food consumption was measured by mixing fluorescent beads comparable in size to bacterial cells with the *E. coli *food source and observing the presence of food within the gut by fluorescence microscopy. Within one hour of exposure, beads were observed in the intestine of 100% of young adult control-fed animals (Figure 3A). The percent of control-fed animals that are consuming beads decreased dramatically with age, however, and by 24 days of age, beads could not detected in the gut lumen of any animals assayed (Figure 3B). Defecation rate also decreased as a function of age and defecation cycles were not observed in control-fed animals older than 24 days (Figure 3C). Thus, as suggested by prior reports measuring pharyngeal pumping [20,32,33], we conclude that consumption of bacterial cells by the control fed population decreases over the first 2–3 weeks of life and largely ceases by 20–24 days of adulthood.

**Figure 4:**
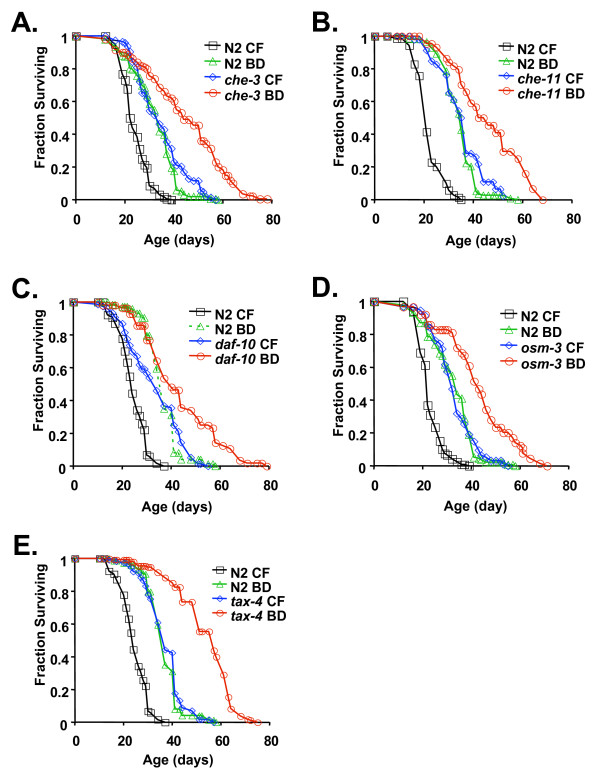
**BD increases life span of select chemosensory mutants**. BD significantly extends the life span of long-lived chemosensory mutants, including (**A**) *che-3(p801) *(3 independent trials) (**B**) *che-11(e1810) *(two trials), (**C**) *daf-10 (e1387) *(2 trials), (**D**) *osm-3(p802) *(2 trials), and (**E**) *tax-4(p678) *(3 trials), compared to control fed (CF) animals. Number of animals examined, mean life spans ± standard error, median life span, and statistical significance are provided in Table 3. Extended statistical analysis (pair-wise comparisons) for each experimental group is provided in Table 4.

Since BD extends life span in animals no longer consuming bacteria (see Figure 1, control fed animals transferred to BD at day 24 of adulthood), we considered the possibility that the bacterial food source produces a longevity-limiting signal that is sensed by the worms. Prior studies demonstrated that some sensory-defective *C. elegans *mutants have increased life span [34-36]. The possibility that dietary cues directly mediate longevity was not examined in these studies, however, and based on physiological assays it was concluded that the long-lived chemosensory mutants were not mimics of DR [35]. Consistent with this conclusion and a recent study using a liquid bacterial dilution DR protocol [37], we observed that BD significantly increased the life span of five long-lived chemosensory mutants examined by Kenyon and colleagues [35]: *che-3(p801)*, *che-11(e1810)*, *daf-10(e1387)*, *osm-3(p802)*, and *tax-4(p678) *(Figure 4A–E). Three of these mutants (*daf-10(e1387)*, *osm-3(p802)*, and *tax-4(p678)*) were previously demonstrated to be at least partially dependent on *daf-16 *[35]; whereas life span extension by BD is *daf-16 *independent [27,28]. BD significantly extends life span in each of these chemosensory mutants compared to control fed animals (Figure 4, Tables 3 and 4). Since the magnitude by which BD increased life span in long-lived chemosensory mutants was comparable to the percent life span increase in N2 animals, we suggest that life span extension from BD is likely to be mediated by a food sensing pathway that is at least partially distinct from the chemosensory mutants examined here.

**Table 3:** Life span extension by BD is additive with known chemosensory pathways.

**Cohort**	**n**	**Mean Survival**	**Median Survival**	**% extension of Median**	***p*-value**
N2 CF	164	24.2 ± 0.4	22.5	-	-
N2 BD	153	32.2 ± 0.7	34.0	51	1.7 × 10^-17^
*che-3(p801) *CF	147	34.3 ± 0.8	34.0	51	2.0 × 10^-21^
*che-3(p801) *BD	168	44.2 ± 1.3	44.0	96	2.6 × 10^-28^

N2 CF	70	22.1 ± 0.6	21.0	-	-
N2 BD	112	34.3 ± 0.7	35.0	67	6.0 × 10^-22^
*che-11(e1810) *CF	45	34.7 ± 1.3	35.0	67	8.6 × 10^-15^
*che-11(e1810) *BD	44	45.9 ± 2.1	44.0	110	2.3 × 10^-16^

N2 CF	117	24.3 ± 0.5	24.0	-	-
N2 BD	74	36.5 ± 0.8	37.0	54	3.7 × 10^-25^
*daf-10 (e1387) *CF	76	32.7 ± 1.3	34.0	42	4.1 × 10^-07^
*daf-10 (e1387) *BD	56	43.1 ± 2.0	40.0	67	1.7 × 10^-17^

N2 CF	119	22.9 ± 0.4	22.0	-	-
N2 BD	131	32.1 ± 0.8	34.0	55	1.1 × 10^-16^
*osm-3(p802) *CF	94	32.4 ± 0.9	32.0	45	5.9 × 10^-18^
*osm-3(p802) *BD	64	42.5 ± 1.8	43.0	95	4.6 × 10^-18^

N2 CF	117	24.3 ± 0.5	24.0	-	-
N2 BD	74	36.5 ± 0.8	37.0	54	3.7 × 10^-23^
*tax-4(p678) *CF	113	36.9 ± 0.7	37.0	54	1.9 × 10^-26^
*tax-4(p678)) *BD	155	53.6 ± 0.9	57.0	138	6.0 × 10^-41^

Life span was measured for a subset of chemosensory mutants, compared to N2, on plates with food (control fed, CF) or on plates without food (bacterial deprivation, BD). The number of animals (n), mean survival (± standard error, SE), median survival, percent extension of the median, and *p*-values are given for each strain and condition. Data shown here are pooled from a minimum of two independent trials. *P*-values were calculated by the Wilcoxon Rank-Sum test using the MATLAB 'ranksum' function.

**Table 4:** Statistical analysis of BD and chemosensory mutant epistasis experiments.

	**N2 CF**	**N2 BD**	***che-3 *CF**	***che-3 *BD**
N2 CF	-	1.7E-17	2.0E-21	2.6E-28
N2 BD	-	-	0.21	3.5E-12
*che-3 *CF	-	-	-	5.6E-09
*che-3 *BD	-	-	-	-

	**N2 CF**	**N2 BD**	***che-11 *CF**	***che-11 *BD**

N2 CF	-	6.0E-22	8.6E-13	2.3E-16
N2 BD	-	-	0.65	1.2E-06
*che-11 *CF	-	-	-	1.9E-04
*che-11 *BD	-	-	-	-

	**N2 CF**	**N2 BD**	***daf-10 *CF**	***daf-10 *BD**

N2 CF	-	3.7E-23	4.1E-07	1.7E-17
N2 BD	-	-	0.08	2.3E-02
*daf-10 *CF	-	-	-	1.8E-04
*daf-10 *BD	-	-	-	-

	**N2 CF**	**N2 BD**	***osm-3 *CF**	***osm-3 *BD**

N2 CF	-	1.1E-16	5.9E-18	4.6E-18
N2 BD	-	-	0.88	4.5E-08
*osm-3 *CF	-	-	-	4.3E-07
*osm-3 *BD	-	-	-	-

	**N2 CF**	**N2 BD**	***tax-4 *CF**	***tax-4 *BD**

N2 CF	-	3.7E-23	1.9E-26	6.0E-41
N2 BD	-	-	0.44	5.3E-21
*tax-4 *CF	-	-	-	1.4E-25
*tax-4 *BD	-	-	-	-

Pair-wise comparisons testing for significant differences in median life span were made for each of the experimental conditions shown in Table 3 and Figure 4. *P*-values were calculated by the Wilcoxon Rank-Sum test using the MATLAB 'ranksum' function.

**Figure 5:**
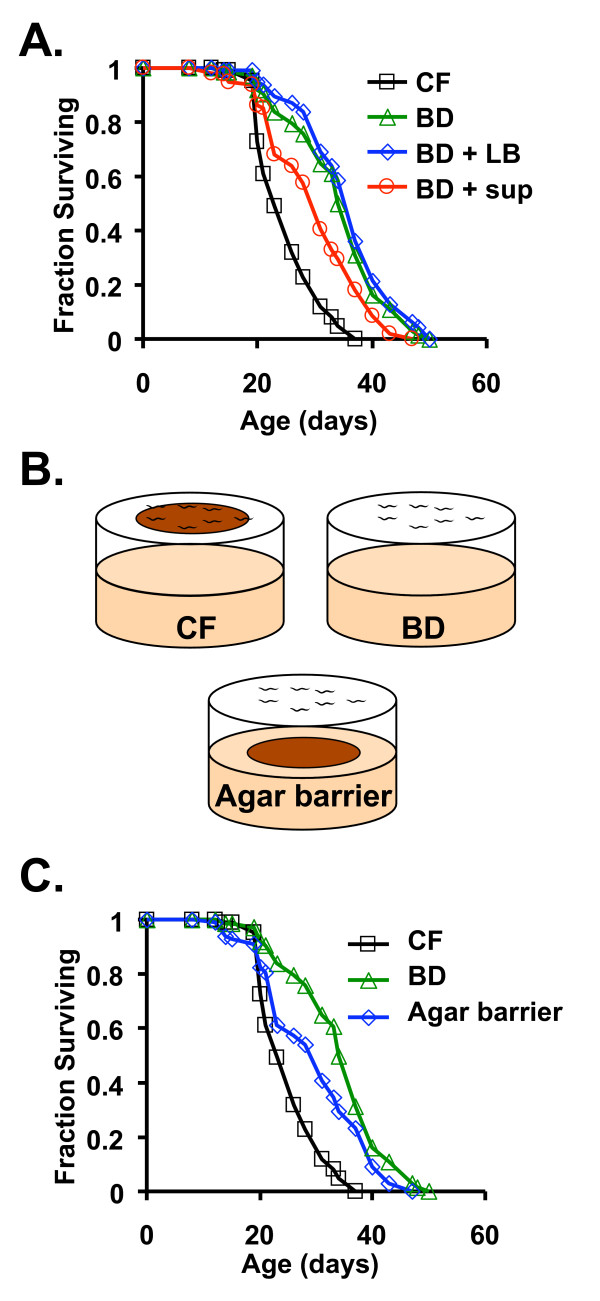
**A diffusible bacterial product suppresses life span extension by bacterial deprivation (BD) in *C. elegans***. (**A**) Animals cultured on plates treated with cell-free supernatant from an overnight culture of *E. coli *OP50 (BD + sup) had decreased survival compared to animals maintained on untreated BD plates or BD plates treated with uninoculated LB (BD + LB). The life span of the treated group was still longer than that of control fed (CF) animals. (**B**) An alternate experimental strategy to exposing animals on BD to bacterial products in the absence of food consumption is to use agar barrier plates, in which a bacterial lawn is separated from the worms by a layer of agar (see Methods for a detailed description). (**C**) Animals cultured on agar barrier plates had decreased survival compared to animals maintained on BD plates with no bacteria present. Number of animals examined, mean life spans ± standard error, median life span, and statistical significance are provided in Table 5A. Extended statistical analysis (pair-wise comparisons) is provided in Table 5B.

To test the hypothesis that decreased sensing of a dietary component underlies a portion of the life span extension from BD, we asked whether cell-free supernatant from an overnight culture of *E. coli *OP50 would influence longevity. Addition of *E. coli *OP50 conditioned supernatant significantly decreased the life span of BD animals (Figure 5A, Table 5). This suppression of BD-induced longevity was not observed when fresh culture media was added to the diet, demonstrating that bacterial metabolism is required for production of the longevity-limiting signal.

**Table 5:** A soluble bacterial product limits life span extension by BD.

**A.**					
**Cohort**	**n**	**Mean Survival**	**Median Survival**	**% extension of Median**	***p*-value**

CF	110	25.1 ± 0.5	23	-	-
BD	74	34.3 ± 0.9	34	48	1.4 × 10^-15^
BD + LB	94	35.9 ± 0.8	37	61	1.4 × 10^-19^
BD + sup	116	30.2 ± 0.8	31	35	2.3 × 10^-07^
Agar Barrier	113	29.6 ± 0.8	31	35	4.0 × 10^-05^

**B.**					

	**CF**	**BD**	**BD + LB**	**BD + sup**	**Agar Barrier**

CF	-	1.4E-13	1.4E-19	2.3E-07	4.0E-05
BD	-	-	2.6E-01	1.0E-03	7.7E-04
BD + LB	-	-	-	2.2E-06	2.6E-06
BD + sup	-	-	-	-	6.4E-01
Agar Barrier	-	-	-	-	-

Life span was measured for N2 on plates with food (control fed, CF), plates without food (bacterial deprivation, BD), BD plates treated with LB (BD + LB) or cell-free supernatant from an *E. coli *OP50 culture (BD + sup), or plates containing a lawn of *E. coli *OP50 beneath the surface of the agar (Agar Barrier). (A) The number of animals (n), mean survival (± standard error, SE), median survival, percent extension of the median, and *p*-values are given for each strain and condition. Data shown here are pooled from two independent trials. *P*-values were calculated by the Wilcoxon Rank-Sum test using the MATLAB 'ranksum' function. (B) Pair-wise comparisons to test for significant differences between median life span were made for each experimental condition and *p-*values are given for each pair.

As a further test of our hypothesis, we determined the longevity of animals that were physically separated from the bacterial food by an agar barrier (Figure 5B). Animals were grown on NGM + OP50 until the 2^nd ^day of adulthood, then transferred to the surface of either control fed, BD, or agar barrier plates. Similar to the effect of adding *E. coli *OP50 conditioned supernatant to the diet, animals maintained on agar barrier plates had a life span that was intermediate between control fed and BD (Figure 5C, Table 5). Thus, we conclude that a diffusible component of the bacterial diet limits longevity, and the absence of this dietary signal accounts for a portion of the life span extension associated with BD.

## Conclusion

**Figure 6:**
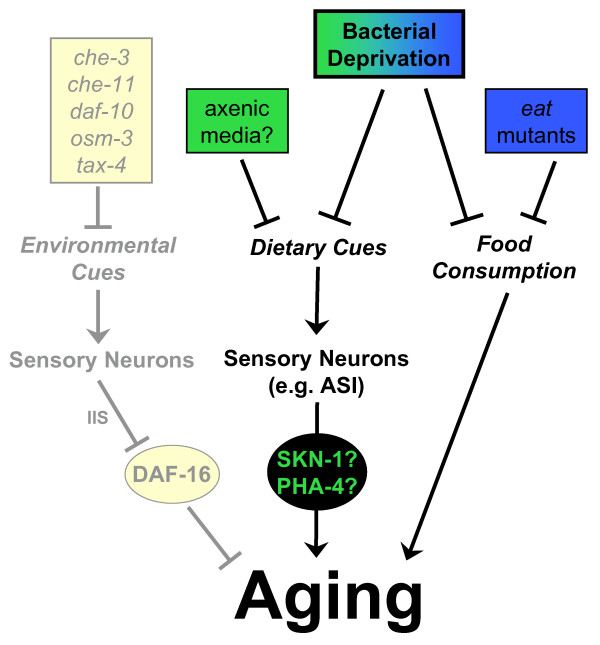
**Bacterial deprivation (BD) extends life span through reduced food consumption and reduced food sensing**. Animals subjected to BD experience calorie restriction through reduced food consumption, similar to genetic models of DR such as mutation of *eat-2*. BD animals are also deprived of a longevity-limiting dietary cue(s) produced by the bacterial food source. Genetic epistasis analysis indicates that BD acts in parallel to long-lived chemosensory mutants (yellow box) that influence life span primarily by altering insulin/IGF-1-like signaling (IIS). Thus, we propose that life span extension from BD is a combination of the effects of calorie restriction and reduced food sensing.

The health and longevity benefits of DR initiated early in life are well established in a variety of organisms [3]. Studies in flies and rodents have indicated that at least some of these benefits can still be attained if DR is initiated during middle age [30,31]. Our work further supports this idea by demonstrating that BD significantly increases survival in *C. elegans *at any point during adulthood, at least up to the median population life span. Importantly, transient BD early in life results in a long-term protective effect, increasing both survival and stress resistance even after return to a control diet. Finally, we report the surprising observation that BD increases survival by a mechanism that is at least partially distinct from reduced food consumption, and involves reduced sensing of a diffusible signal produced by the bacterial component of the diet. Based on these observations, we propose that DR increases life span through two distinct mechanisms: calorie restriction and reduced food sensing (Figure 6).

The idea that dietary cues could influence nematode longevity via sensory signaling is supported by prior observations. *C. elegans *is attracted to both soluble and volatile compounds produced by the bacterial food source, and animals can sense and respond to food through chemotaxis [38]. Although muscle tissue undergoes age-related deterioration, the nervous system remains remarkably intact in worms of advanced age, [39]. This is consistent with our model that BD extends life span in worms of advanced age, not because of reduced food consumption, but because of the loss of a life span-limiting signal produced by the bacteria and sensed by the worms. In addition, several mutants defective in chemosensation are long-lived. The additive life span extension from combining long-lived chemosensory mutants with BD is consistent with our model, as these mutants were previously shown not to be genetic mimics of DR [35]. Indeed, since life span extension in chemosensory mutants is largely dependent on *daf-16 *[35], and multiple epistasis studies have placed DR and *daf-16 *in different genetic pathways, an additive life span extension is the predicted outcome of these experiments.

How can a sensory mechanism of longevity determination by BD be reconciled with other models of DR in *C. elegans*? Mutation of *eat-2 *causes a pharyngeal pumping defect that results in reduced food consumption [12,13], but has not been shown to cause sensory defects. Thus, the life span extension from mutation of *eat-2 *is likely due to a reduction in caloric intake. Interestingly, BD increases life span to a greater extent than mutation of *eat-2*, and *eat-2 *mutants subjected to BD are not longer-lived than N2 animals subjected to BD [27,28]. One interpretation of these data is that reduced food intake (e.g. in the *eat-2 *mutant) is sufficient to increase life span, while reduced food intake coupled with reduced food sensing (e.g. in the case of BD) leads to an even greater life span extension. Axenic culture is somewhat more complicated because, although the animals are maintained in the absence of food, as they are in BD, the growth media is very nutrient rich (compared to NGM) and contains additional signals that may be sensed by *C. elegans*. *eat-2 *mutants subjected to axenic culture live longer than N2 animals on axenic growth [23], suggesting that mutation of *eat-2 *and axenic growth are not equivalent methods of DR.

Previous studies have demonstrated a role for the bacterial food source in life span determination – through bacterial pathogenesis. A number of bacterial strains have been shown to be pathogenic in *C. elegans *[40], and worms cultured on a non-pathogenic species, *B. subtilis*, were shown to have extended longevity compared to those cultured on the normal food source, *E. coli *OP50 [41]. OP50 has also been shown to affect life span through bacterial proliferation in the gut [42]. In our studies, we maintain worms on UV-irradiated OP50 as a food source to eliminate bacterial pathogenesis as a complicating factor in life span analysis. However, we do find that a soluble product made by OP50 partially suppresses the life span extension by BD, independent of bacterial proliferation, supporting our model that reduced food sensing contributes to the longevity observed in animals subjected to BD. Formally, it is possible that the bacteria produce a toxin that is life span-limiting, independent of the pathogenic effects of bacterial proliferation in the gut. This does not change the interpretation of our data, however, which is that a diffusible, cell-free component of the diet can limit the life span extension from BD.

Recent studies in the fruit fly have indicated that a volatile component of the diet can also suppress life span extension from DR in that organism [43]. Thus, modulation of longevity through food sensing may represent an evolutionarily conserved mechanism linking diet and aging. It will be of interest to determine which are the precise chemical cues in the diet that influence longevity in each of these organisms. At this time, there is little direct evidence that food sensing modulates aging in mammals; however, it is reasonable to speculate that food could induce relevant physiological changes through olfactory or taste cues, independent of food consumption. This raises the intriguing possibility that one class of DR mimetics may be achieved by interfering with food sensing, but without requiring reduced caloric intake.

## Methods

### Worm Strains and Maintenance

*C. elegans *were propagated at 20°C on NGM seeded with *E. coli *OP50 in 5 cm Petri dishes. Unless otherwise stated, *E. coli *OP50 cultures used for seeding plates were inoculated from a single bacterial colony in Luria broth (LB) and allowed to grow overnight at 37°C, then concentrated 5-fold before seeding on NGM plates containing 100 μg/ml ampicillin. The bacterial food source for all experiments was UV-killed after seeding in a Stratalinker (9999 J/m^2^), with UV-killing verified by failure to form colonies upon streaking to LB plates. Plates lacking food were similarly treated with ampicillin and UV.

### Life span assays

Life span assays were initiated by allowing young adult hermaphrodites to lay eggs for 4–6 hours on NGM containing ampicillin, seeded with UV-killed OP50. At L4, animals were transferred to fresh NGM + UV-killed OP50 supplemented with ampicillin and 50 μM 5-fluorodeoxyuridine (FUDR) to prevent progeny from hatching. Unless otherwise stated, at the 4^th ^day of adulthood animals were transferred to experimental media: either NGM + UV-killed OP50 + ampicillin/FUDR (control-fed media) or NGM + ampicillin/FUDR (BD media). In some experiments, tetracycline was added topically to plates to eliminate contaminants. Fed animals were transferred to fresh plates every three days for the first two weeks of each life span experiment and then as necessary to prevent depletion of the food source. The viability of each animal was determined every 2–3 days by assaying for movement in response to agitation of the plate or gentle probing with a platinum wire. Animals that crawled off the NGM agar surface and failed to return were excluded from subsequent analyses. All of the experiments shown were repeated at least 3 times, with similar results.

For experiments testing the effect of soluble bacterial products on life span, plates were prepared that had a bacterial lawn beneath the agar surface. *E. coli *OP50 was seeded on a thin NGM agar pad (5 ml) before adding a second layer of NGM agar (5 ml). Total volume of NGM agar was the same for experimental and control groups (10 ml). Control plates with food on the surface and control BD plates were made in the same way, without a bacterial lawn in between agar layers. In experiments testing cell-free supernatant from *E. coli *OP50 culture, plates were prepared by adding 100 μl of filter-sterilized cell-free supernatant (the same volume used for seeding control-fed plates).

### Thermotolerance Assays

Animals were maintained at 20°C until beginning the thermotolerance assay. Response to heat stress was assayed by measuring viability over time at 38°C. Viability was determined by removing one plate at a time (~20 animals per plate) from the incubator and assaying for movement in response to gentle prodding. Each experiment included a minimum of two plates per condition and we performed three independent trials.

### Defecation Assays

For defecation assays, single animals were transferred to fresh NGM plates seeded with UV-killed OP50 + ampicillin/FUDR and allowed to recover from transfer for one hour prior to the assay. Individual animals were observed under a dissecting microscope for a ten-minute period and each posterior body contraction and expulsion were recorded. Completed defecation cycles were scored when both a posterior body contraction and expulsion were observed.

### Feeding Assays

Consumption of fluorescent beads was measured as previously described [44]. Briefly, NGM plates seeded with UV-killed OP50 + ampicillin/FUDR were treated with 100 μl of fluorescent beads diluted 1:20 in M9 buffer and allowed to dry. Fluorescent beads used in these assays were Fluoresbrite polychromatic red microspheres (0.5 μm) from Polysciences, Inc. For each age and condition assayed, ten animals were transferred to plates treated with beads and allowed to feed for an hour. Animals were then briefly transferred to plates without beads to remove excess beads from the cuticle and then transferred to Teflon-coated slides in M9 buffer with 25 mM sodium azide for microscopy. Animals with detectable beads in the gut lumen (by fluorescent microscopy) were scored as positives. Images were generated with a Canon Powershot S31S digital camera connected to the eyepiece of a Zeiss SteREO Lumar.V12 microscope.

### Statistical analysis

A Wilcoxon Rank-Sum test was used (MATLAB 'ranksum' function) to determine whether median survival differed between groups in all life span and thermotolerance experiments. A *p*-value < 0.05 was considered statistically significant.

## Authors' contributions

EDS participated in the conception and design of the study, coordinated the data acquisition and analysis, and drafted the manuscript. TLK participated in the conception and design of the study, data acquisition and analysis. BTL contributed to experimental design, data acquisition, and data analysis of feeding and defecation assays. JS contributed to experimental design, data acquisition, and data analysis of thermotolerance assays. KLW contributed to experimental design, data acquisition, and data analysis of life span experiments. BKK participated in the conception and design of the study, and drafted the manuscript. MKK conceived of the study, participated in its design and coordination, and drafted the manuscript. All authors read and approved the final manuscript.
